# [^18^F]FDG and [^68^Ga]Ga-FAPI-04–Directed Imaging for Outcome Prediction in Patients with High-Grade Neuroendocrine Neoplasms

**DOI:** 10.2967/jnumed.124.268288

**Published:** 2024-12

**Authors:** Kerstin Michalski, Aleksander Kosmala, Philipp E. Hartrampf, Marieke Heinrich, Sebastian E. Serfling, Wiebke Schlötelburg, Andreas K. Buck, Alexander Meining, Rudolf A. Werner, Alexander Weich

**Affiliations:** 1 Department of Nuclear Medicine, University Hospital Würzburg, Würzburg, Germany;; 2 NET-Zentrum Würzburg, European Neuroendocrine Tumor Society Center of Excellence, University Hospital Würzburg, Würzburg, Germany;; 3 Department of Internal Medicine II, Gastroenterology, University Hospital Würzburg, Würzburg, Germany;; 4 Department of Nuclear Medicine, Clinic for Radiology and Nuclear Medicine, University Hospital, Goethe University Frankfurt, Frankfurt, Germany; and; 5 Russell H. Morgan Department of Radiology and Radiological Sciences, Johns Hopkins School of Medicine, Baltimore, Maryland

**Keywords:** dual-tracer PET/CT, FAPI and FDG, discordant lesions, neuroendocrine neoplasms, outcome prediction

## Abstract

We aimed to quantitatively investigate the prognostic value of PET-based biomarkers on [^18^F]FDG and [^68^Ga]Ga-fibroblast activation protein inhibitor (FAPI)-04 PET/CT in patients with highly aggressive neuroendocrine neoplasms (NENs) and to compare the visually assessed differences in uptake on both examinations with progression-free survival (PFS). **Methods:** In this single-center retrospective analysis, 20 patients with high-grade NENs had undergone [^18^F]FDG and [^68^Ga]Ga-FAPI-04 PET. Both PET scans were visually compared, and the presence of [^18^F]FDG-positive, [^68^Ga]Ga-FAPI-04–negative (FDG+/FAPI−) lesions was noted. In addition, we assessed maximum, peak, and mean SUV; tumor volume (TV); and total lesion uptake (TLU = TV × SUV_mean_) for both radiotracers using a 40% lesion-based threshold. The results of quantitative and visual analysis were correlated with PFS using log-rank analysis or univariate Cox regression. PFS was defined radiographically using RECIST 1.1., clinically using signs of disease progression, or as death. **Results:** Most primary tumors were located in the gastrointestinal tract (13/20 patients, 65%) or were cancer of unknown primary (5/20 patients, 25%). FDG+/FAPI− lesions were found in 9 of 20 patients (45%). Patients with FDG+/FAPI− lesions had a significantly decreased PFS of 4 mo, compared with 9 mo for patients without FDG+/FAPI− metastases (*P* = 0.0063 [log-rank test]; hazard ratio [HR], 5.637; 95% CI 1.619–26.16; *P* = 0.0110 [univariate Cox regression]). On univariate analysis, a significant correlation was also found between PFS and TV for both radiotracers ([^18^F]FDG: mean TV, 258 ± 588 cm^3^; HR, 1.024 [per 10 cm^3^]; 95% CI, 1.007–1.046; *P* = 0.0204) ([^68^Ga]Ga-FAPI-04: mean TV, 130 ± 192 cm^3^; HR, 1.032 [per 10 cm^3^]; 95% CI, 1.001–1.062; *P* = 0.0277) and TLU on [^18^F]FDG PET (mean TLU, 1,931 ± 4,248 cm^3^; HR, 1.004 [per 10 cm^3^]; 95% CI, 1.001–1.007; *P* = 0.0135). **Conclusion:** The presence of discordant FDG+/FAPI− lesions is associated with a significantly shorter PFS, which might indicate more aggressive disease prone to early progression. Dual-tracer PET/CT of patients with highly aggressive NENs could help guide treatment decisions or identify high-risk lesions for additional local therapeutic approaches.

## Visual Abstract

**Figure:**
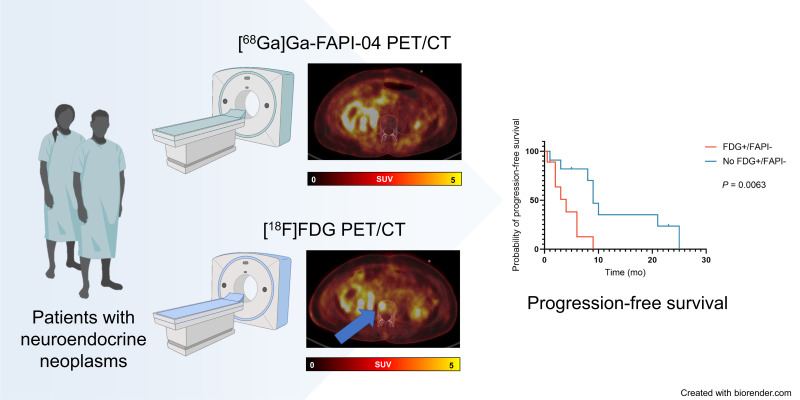

Neuroendocrine neoplasms (NENs) are a rare heterogeneous group of tumors with a predominantly neuroendocrine differentiation that may occur in many different organ systems but occurs most commonly in the gastrointestinal tract and the lung. The clinical course varies considerably between patients, depending on the degree of dedifferentiation, which is reflected by tumor grade. Grading depends on histomorphologic features as well as proliferation index (Ki-67) and segregates NENs into differentiated neuroendocrine tumors (NETs) and poorly differentiated neuroendocrine carcinomas (NECs). Differentiated NETs can be divided into low-grade (G1; Ki-67, 1%–2%), intermediate-grade (G2; Ki-67, 3%–20%), and high-grade (G3; Ki-67, >20%) types (1*,*^2^). NECs are always considered high-grade and poorly differentiated, typically displaying a Ki-67 of more than 70%. In rare cases, NENs (well-differentiated and poorly differentiated) can occur as mixed NENs and non-NENs (MiNENs) that display components of neuroendocrine as well as adenoid differentiation. In most cases, the neuroendocrine component consists of a NEC and the tumor is aggressive (1).

Well-differentiated NENs show high expression of somatostatin receptors (SSTRs). Thus, they can be studied and treated using a theranostic approach with radiolabeled DOTA-peptides such as [^68^Ga]Ga/[^177^Lu]Lu-DOTATATE (3). [^18^F]FDG PET/CT is used in patients with poorly differentiated NENs and in high-grade NENs before SSTR-directed radiopharmaceutical therapy to exclude possible SSTR-negative lesions or to detect more aggressive subclones that would be pivotal to the patient’s prognosis. In this sense, detection of these lesions could guide local therapeutic approaches or lead to an adjustment of systemic treatment. This tumor heterogeneity cannot be detected by a core biopsy of a single tumor lesion but needs molecular whole-body tumor assessment that can be provided by PET (4). Regardless of the tumor grade, [^18^F]FDG PET also predicts an aggressive course of disease and a poorer prognosis (5*,*^6^).

Cancer-associated fibroblasts are another possible molecular target for PET in NENs. These fibroblasts enhance tumor invasion, tumorigenesis, and metastasis by promoting angiogenesis, epithelial–mesenchymal transition, and proliferation or secretion of soluble factors in many solid tumors of the digestive system (7). Fibroblast activation protein (FAP) is overexpressed on cancer-associated fibroblasts and can be targeted by FAP inhibitor (FAPI) PET (8). FAP is especially overexpressed by epithelial carcinomas but—to a much lesser extent—might also be found on tumors with neuroendocrine differentiation (3). Kratochwil et al. described high in vivo FAP expression imaged by PET in different adenocarcinomas and rather moderate uptake in a small number of NETs (9). In a previous study, our group showed that [^68^Ga]Ga-FAPI-04 is a useful staging method for highly proliferative NENs (G3 NETs or NECs) of the gastroenteropancreatic system (10). We therefore aimed to investigate the use of dual-tracer [^68^Ga]Ga-FAPI-04 and [^18^F]FDG PET/CT in assessing tumor heterogeneity in patients with NENs and the prognostic value of this assessment.

## MATERIALS AND METHODS

### Patient Cohort

Patients were included in this retrospective single-center study if they had a histologically proven highly aggressive NEN (G3 NET, NEC, or MiNEN) and in-house assessment of [^68^Ga]Ga-FAPI-04 and [^18^F]FDG PET/CT. The interval between scans had to be no more than 3 mo, without intermittent tumor-specific therapy. Patients were excluded if follow-up data were missing. Patients underwent dual-tracer PET/CT on a compassionate-use basis by the treating physicians to detect possible heterogeneous or aggressive tumor lesions that might change the tumor grade. Written informed consent was obtained from all patients. The need for further approval was waived by the local institutional review board because of the retrospective nature of the study (study 20230721 01). Parts of the cohort have been published before (10) but without focusing on the impact of dual-tracer PET/CT.

### Image Acquisition

Synthesis and labeling of [^68^Ga]Ga-FAPI-04 was performed as described previously (11). [^18^F]FDG was produced using a GE FASTlab synthesis module (GE HealthCare). The radiochemical purity of [^18^F]FDG was at least 95%. Patients had to fast for at least 6 h, and the blood glucose level had to be lower than 180 mg/dL before [^18^F]FDG injection. The mean injected activity of [^18^F]FDG was 235 MBq (range, 154–316 MBq). At 1 h after injection of both tracers, patients underwent whole-body PET (from vertex to mid thigh) with a scan duration of 2 min per bed position. A Biograph mCT device (64 or 128; Siemens Healthineers) was used with or without intravenous contrast material (activated automatic tube current modulation; reference mAs, 35 mAs for low-dose scans and 160 mAs for full-dose scans; tube voltage, 120 keV/100 keV [mCT 64/mCT 128]; pitch, 1.4/0.8 [mCT 64/mCT 128]; collimation, 64/128 × 0.6 mm; rotation time, 0.5 s; reconstructed axial slice thickness, 3.0–5.0 mm), and PET images were reconstructed using standard parameters (3-dimensional mode; matrix, 200 × 200; iterations, 3; subsets, 24 [mCT 64]/21 [mCT 128]; gaussian filtering, 2.0 mm).

### Image Analysis

All PET/CT scans were quantitatively analyzed by one board-certified radiologist using Syngo.via (Siemens Healthineers) with a 40% lesion-specific threshold as already published by our group (12–15). Maximum, peak, and mean SUV; tumor volume (TV); and total lesion uptake (TLU = TV × SUV_mean_) for both radiotracers were noted. Visual assessment of discordant [^18^F]FDG-positive, [^68^Ga]Ga-FAPI-04–negative (FDG+/FAPI−) lesions was done in consensus by one board-certified radiologist and one board-certified nuclear medicine specialist, both with more than 5 y of PET/CT reading experience. FDG+/FAPI− was defined as visually higher uptake than in the liver on [^18^F]FDG PET and lack of uptake compared with local background on [^68^Ga]Ga-FAPI-04 PET. All lesions that were visually assessed as discordant had to show a higher SUV_peak_ and tumor-to-background ratio (SUV_peak_ of tumor lesions/SUV_mean_ of the descending aorta). In addition, the volume of FDG+/FAPI− lesions on [^18^F]FDG PET was noted.

### Statistical Analysis

Prism version 9.3.1 (GraphPad Software) was used for statistical analysis. Descriptive data are presented as mean ± SD and range. Quantitative data from both PET scans were compared using the Wilcoxon matched-pairs signed rank test. Progression data were analyzed by Kaplan–Meier curves and log-rank comparison. Univariate Cox proportional-hazards regression for continuous variables was undertaken. Progression-free survival (PFS) was defined radiographically using RECIST 1.1., clinically using signs of disease progression as assessed by the treating physicians, or as death from any cause. Radiographic PFS was assessed by one board-certified radiologist using RECIST 1.1 (16). Calculation of PFS started with the day of the [^68^Ga]Ga-FAPI-04 PET/CT scan and continued until progression.

## RESULTS

### Patient Cohort

This study included 20 patients (11 men, 9 women) with highly aggressive NENs (G3 NETs, NECs, and MiNENs) who underwent [^68^Ga]Ga-FAPI-04 and [^18^F]FDG PET/CT between July 2020 and March 2023. The mean time between the scans was 10 ± 14 d (range, 1–63 d). The mean patient age on the day of [^68^Ga]Ga-FAPI-04 PET/CT was 60 ± 10 y (range, 38–81 y). The mean Ki-67 was 69% ± 24% (range, 14%–90%), and most patients had NECs (9/20 patients, 45%) or MiNENs (6/20 patients, 30%). Detailed patient characteristics are given in Table 1.

**TABLE 1.** Patient Characteristics

Patient no.	Age (y)	Sex	Primary tumor	Histology	Ki-67 (%)	Indication	Previous therapy	Therapy until progression
1	59	F	Colon	MiNEN	80	Primary staging	—	Carboplatin/etoposide
2	63	F	Colon	MiNEN	>85	Primary staging	—	Carboplatin/etoposide
3	69	M	CUP	NEC	>80	Primary staging	—	Radiotherapy (bone metastasis)
4	81	M	CUP	NEC	>80	Primary staging	—	Carboplatin/etoposide/atezolizumab
5	56	M	Pancreas	G3	70	Restaging	Cisplatin/etoposide	Octreotide, PRRT
6	38	F	Pancreas	G3	60	Primary staging	—	Octreotide, PRRT
7	75	F	Small intestine	G3	20	Restaging	Surgery, TACE, everolimus	Temozolomide/capecitabine
8	57	M	CUP	NEC	Unknown	Primary staging	—	Carboplatin/etoposide
9	42	M	Pancreas	G3	40	Primary staging	—	Streptozocin/5-fluorouracil
10	53	M	Stomach	MiNEN	90	Restaging	Cisplatin/etoposide	Surgery, cisplatin/etoposide
11	49	F	Breast	NEC	90	Primary staging	—	Surgery, adjuvant radiotherapy
12	64	M	Bladder	NEC	90	Primary staging	—	Carboplatin/etoposide
13	66	F	CUP	NEC	70	Primary staging	—	Carboplatin/etoposide
14	60	F	Colon	MiNEN	90	Primary staging	—	Carboplatin/etoposide
15	59	M	Pancreas	NEC	14	Restaging	Octreotide, PRRT, TACE, FOLFOX	Carboplatin/etoposide
16	53	M	Pancreas	G3	40	Restaging	Octreotide, streptozocin/ 5-fluorouracil, temozolomide/capecitabine	Octreotide, PRRT
17	68	M	Stomach	NEC	90	Primary staging	—	Carboplatin/etoposide
18	68	M	Pancreas	MiNEN	80	Primary staging	—	FOLFIRINOX
19	67	F	CUP	NEC	70	Primary staging	—	Surgery, adjuvant radiotherapy, carboplatin/etoposide/atezolizumab
20	58	F	Colon	MiNEN	75	Restaging	Cisplatin/etoposide	Cisplatin/etoposide

CUP = cancer of unknown primary; PRRT = peptide receptor radionuclide therapy; TACE = transarterial chemoembolization; FOLFOX = folinic acid, fluorouracil, and oxaliplatin; FOLFIRINOX = folinic acid, fluorouracil, irinotecan hydrochloride, and oxaliplatin.

### PFS-Prognostic FDG+/FAPI− Lesion Discordance

**FIGURE 1.**
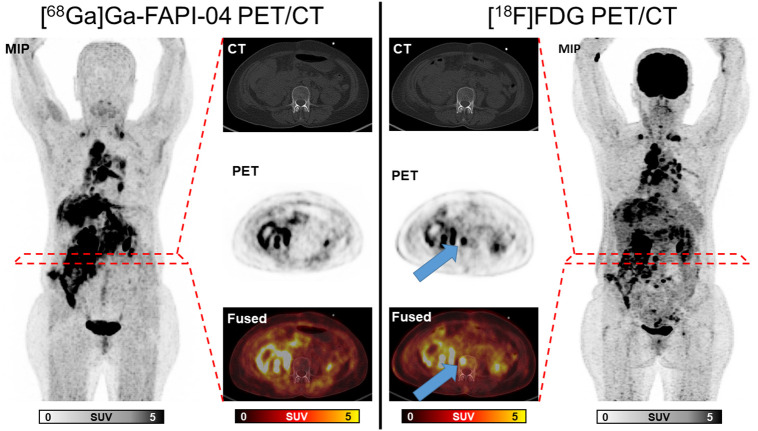
Maximum-intensity projection (MIP), CT, PET, and fused images of [^68^Ga]Ga-FAPI-04 PET/CT and [^18^F]FDG PET/CT of patient 2. Images show concordant high uptake in multiple lymph node metastases and advanced peritoneal carcinomatosis. In addition, [^18^F]FDG PET shows osseous metastases not seen on [^68^Ga]Ga-FAPI-04 PET (arrows). Time difference between scans was 1 d.

Metastatic sites were found in 18 of 20 patients (90%) in the liver (*n* = 12), lymph nodes (*n* = 12), bones (*n* = 8), lungs (*n* = 3), and peritoneum (*n* = 3). FDG+/FAPI− lesions were found in 9 of 20 patients (45%, an example patient is shown in Fig. 1), with the following organ systems affected: liver (*n* = 4), bones (*n* = 3), and lymph nodes (*n* = 2). Discordant FDG+/FAPI− lesions were found in patients with all included histologic subtypes: NECs (*n* = 5), MiNENs (*n* = 2), and G3 NETs (*n* = 2). A detailed evaluation of FDG+/FAPI− lesions can be found in Supplemental Table 1 (supplemental materials are available at http://jnm.snmjournals.org). The mean discordant volume on [^18^F]FDG PET was 11.6 ± 8.7 cm^3^ (0.8–26.7 cm^3^), which consists of 13.4% ± 14.9% (range, 1%–47%) of the total volume on [^18^F]FDG PET. In 2 of 20 patients (10%), reverse-discordant FDG+/FAPI− lesions were found in the bones.

**FIGURE 2.**
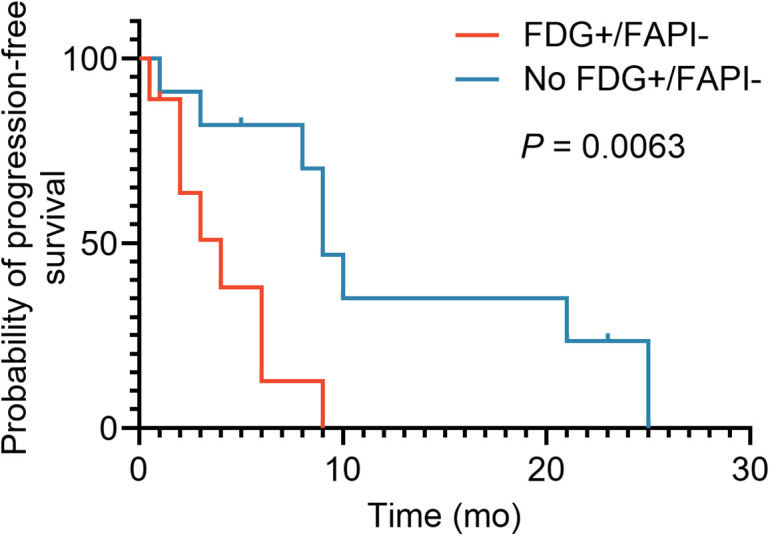
Kaplan–Meier curves of PFS of patients with (*n* = 9, red line) or without (*n* = 11, blue line) discordant FDG+/FAPI− lesions. Patients with discordant lesions showed significantly shorter median PFS (4 mo) than patients without (9 mo, *P* = 0.0063).

The median PFS of all patients was 8 mo, with 16 patients showing progression. Patients with FDG+/FAPI− lesions had a significantly shorter PFS (4 mo) than did patients without FDG+/FAPI− metastases (9 mo) (*P* = 0.0063; Fig. 2).

On quantitative analysis, no significant differences in extracted values were found between the 2 radiotracers in any patients (*P* > 0.2): [^18^F]FDG TV, 258 ± 588 cm^3^ (range, 1–2,604 cm^3^); [^68^Ga]Ga-FAPI-04 TV, 130 ± 192 cm^3^ (range, 1–735 cm^3^); [^18^F]FDG TLU, 1,931 ± 4,248 cm^3^ (range, 1–18,682 cm^3^); [^68^Ga]Ga-FAPI-04 TLU, 1,024 ± 1,644 cm^3^ (range, 1–6,534 cm^3^); [^18^F]FDG SUV_mean_, 6.7 ± 3.6 (range, 1.7–15.2); [^68^Ga]Ga-FAPI-04 SUV_mean_, 6.2 ± 3.2 (range, 1.3–15.4); [^18^F]FDG SUV_max_, 12.3 ± 6.1 (range, 1.7–27.7); [^68^Ga]Ga-FAPI-04 SUV_max_, 11.5 ± 6.4 (range, 1.3–29.3); [^18^F]FDG SUV_peak_, 7.9 ± 3.9 (range, 1.7–17.8); and [^68^Ga]Ga-FAPI-04 SUV_peak_, 7.1 ± 4.7 (range, 1.3–22.2). On univariate analysis, a significant correlation was found between PFS and the presence of FDG+/FAPI− lesions, TV for both radiotracers, and TLU on [^18^F]FDG PET. The other quantitative parameters failed to reach significance (Table 2).

**TABLE 2.** Univariate Analysis

Parameter	Hazard ratio	95% CI	*P*
[^18^F]FDG TV (per 10 cm^3^)	1.024	1.007–1.046	0.0204
[^18^F]FDG TLU (per 10 cm^3^)	1.004	1.001–1.007	0.0135
[^18^F]FDG SUV_mean_	1.114	0.9721–1.262	0.0961
[^18^F]FDG SUV_max_	1.063	0.9799–1.145	0.1190
[^18^F]FDG SUV_peak_	1.100	0.9644–1.248	0.1419
[^68^Ga]Ga-FAPI-04 TV (per 10 cm^3^)	1.032	1.001–1.062	0.0277
[^68^Ga]Ga-FAPI-04 TLU (per 10 cm^3^)	1.001	1.002–1.003	0.5262
[^68^Ga]Ga-FAPI-04 SUV_mean_	1.028	0.9040–1.153	0.6463
[^68^Ga]Ga-FAPI-04 SUV_max_	1.020	0.9579–1.081	0.5101
[^68^Ga]Ga-FAPI-04 SUV_peak_	1.015	0.9278–1.093	0.7173
FDG+/FAPI− lesions	5.673	1.619–26.16	0.0110
Histology (G3 NET, NEC, MiNEN)	1.386	0.7465–2.609	0.2977
Age (y)	1.057	1.003–1.121	0.0477

## DISCUSSION

To our knowledge, this study was the first to evaluate the prognostic impact of dual-tracer [^18^F]FDG and [^68^Ga]Ga-FAPI-04 PET/CT in patients with various highly aggressive NENs, including rare cases of MiNENs. We found discordant FDG+/FAPI− lesions in a substantial fraction of patients (45%), reflecting tumor heterogeneity. These patients showed a significantly reduced PFS of 4 mo, compared with the 9 mo in patients without FDG+/FAPI− lesions (*P* = 0.0063). Interestingly, discordant FDG+/FAPI− lesions were found in patients with different histologic grades. Whole-body tumor assessment using PET apparently adds molecular findings to pathologic examination, although the relative proportion of the discordant volume is rather small.

Dendl et al. examined 55 patients with rare tumor entities using [^68^Ga]Ga-FAPI-04 PET/CT. Among these, 4 patients had neuroendocrine cancer and showed modest uptake, even though patients with epithelial carcinoma appeared to be more suitable for [^68^Ga]Ga-FAPI-04 PET/CT (17). However, uptake was not compared with [^18^F]FDG in this analysis. In general, [^68^Ga]Ga-FAPI-04 PET offers a high tumor-to-background ratio, which enables, especially, a better depiction of liver metastases (18*,*^19^).

Discordant lesions on [^18^F]FDG PET and PET targeting the prostate-specific membrane antigen (PSMA) have already been widely discussed in patients with metastasized castration-resistant prostate cancer (20–26). Detection of [^18^F]FDG-positive/PSMA-negative lesions was a significant negative predictor for overall survival in patients before PSMA-targeted radiopharmaceutical therapy in a retrospective analysis (21). However, PSMA is the target used for endoradiotherapy in a theranostic approach, whereas the patients included in this study were not treated with FAP-targeted endoradiotherapy and the reduced outcome cannot be explained by reduced uptake of the therapeutic agent. Of note, FAP-targeted radiopharmaceutical therapy is a possible therapeutic option and has shown promising results in other tumor entities (27). FAP-targeted radiopharmaceutical therapy could broaden therapeutic options in patients with low or heterogeneous SSTR expression on SSTR-directed PET/CT. In this sense, our results indicate the need for a dual-tracer approach to exclude FAP-negative lesions that cannot be targeted by endoradiotherapy.

Chan et al. investigated dual-tracer PET/CT in patients with NENs using [^18^F]FDG and SSTR-directed PET/CT. Both in a retrospective monocentric analysis with 62 patients and in a retrospective multicenter study with 319 patients, discordant [^18^F]FDG-positive but SSTR-negative lesions were a negative predictor for overall survival in patients with gastroenteropancreatic NENs (28*,*^29^). In a recent analysis, Chan et al. examined the discrepancies of TV on [^18^F]FDG and [^68^Ga]Ga-DOTATATE PET/CT in a retrospective multicenter analysis. The authors included 44 patients with gastroenteropancreatic NENs and found a significantly reduced overall survival for patients with a highly discordant TV compared with a less discordant TV (30). To our knowledge, our study is the first to describe discordant lesions on [^18^F]FDG and [^68^Ga]Ga-FAPI-04 PET/CT for patients with NENs. In analogy to discordant lesions on [^18^F]FDG and SSTR-directed PET, a soley high glycolytic index in some lesions on molecular imaging appears to be predictive of patient outcome. Interestingly, a high glucose metabolism (reflected by SUV_max_/SUV_peak_) was not relevant for outcome prediction but for the presence of discordant lesions.

This retrospective single-center analysis had several limitations. Because of the rarity of NENs, our study included only 20 patients with heterogeneous primary tumors. However, this was, to our knowledge, the largest cohort of patients with highly aggressive NENs examined with [^68^Ga]Ga-FAPI-04 and [^18^F]FDG PET/CT. We did not investigate the diagnostic potential of these tracers for NENs, as the focus of this analysis was to elucidate tumor heterogeneity and its prognostic impact. We used PFS for outcome definition because most patients underwent dual-tracer PET/CT as part of their primary staging, resulting in a long follow-up time until death. Last, the interval between scans could be up to 3 mo as an inclusion criterion, potentially leading to false-positive discordant FDG+/FAPI− lesions. However, the interval between discordant PET scans was short (range, 1–21 d; mean, 9 d). Hence, the presence of discordant lesions was not due to progression between the scans.

## CONCLUSION

The presence of discordant FDG+/FAPI− lesions is associated with a significantly shorter PFS in patients with high-grade NENs, possibly indicating more aggressive disease prone to early progression. Of note, the impact of in vivo visualization of this tumor heterogeneity using dual-tracer PET/CT was not related to tumor grade. Thus, the combination of [^68^Ga]Ga-FAPI-04 and [^18^F]FDG PET/CT in patients with NENs could help guide treatment decisions or identify lesions for additional stereotactic approaches.

## DISCLOSURE

This work was supported by the Bavarian Cancer Research Center (personal grant to Kerstin Michalski) and by the Interdisciplinary Center of Clinical Research (IZKF), University Hospital of Wuerzburg (grant Z-2/91 to Wiebke Schlötelburg and grant Z-3BC/08 to Kerstin Michalski). No other potential conflict of interest relevant to this article was reported.

## Notes

KEY POINTS **QUESTION:** Is tumor heterogeneity as assessed with [^18^F]FDG and [^68^Ga]Ga-FAPI-04 PET/CT relevant to outcome in patients with highly aggressive NENs? **PERTINENT FINDINGS:** In this retrospective, single-center study, patients with FDG+/FAPI− lesions (9/20) had a significantly shorter PFS. **IMPLICATIONS FOR PATIENT CARE:** Dual-tracer PET/CT of patients with high-grade NENs could help guide treatment decisions or identify high-risk lesions for additional local therapeutic approaches.
